# Dynamics of Digital Support Platform Use Among Family Caregivers of Children With Disabilities: Longitudinal Study

**DOI:** 10.2196/92481

**Published:** 2026-09-23

**Authors:** Xiaojiao Yuan, Xi Zhao, Meng Zhang, Yurou Duan, Yizhong Shui

**Affiliations:** 1 School of Education and Psychology, Southwest Minzu University, Chengdu, China; 2 Health Care and Nursing College, Southwest Jiaotong University Hope College, Chengdu, China; 3 School of Economics and Management, Chengdu Normal University, No. 99 East Haike Road, Wenjiang District, Chengdu, Sichuan, China, 86 02865791142

**Keywords:** digital support, family caregivers, children with disabilities, adoption, engagement

## Abstract

**Background:**

Family caregivers of children with disabilities frequently experience high levels of stress and psychological burden. Digital support platforms offer accessible channels for information and emotional support, but their long-term use patterns remain underexplored.

**Objective:**

This study explored the longitudinal patterns of digital support platform use among family caregivers in China, with a focus on factors potentially associated with initial adoption and continued engagement.

**Methods:**

A 3-wave survey was conducted over 6 months with 240 primary caregivers of children with disabilities in China. A 2-part latent growth curve model was used to jointly model adoption (use vs nonuse) and engagement (use frequency among users). Key demographic, psychosocial, and child-related factors were examined as predictors.

**Results:**

Adoption increased significantly over time (probit slope=0.54, SE 0.16; *P*=.001). Higher baseline adoption was associated with male sex (β=.19; *P*=.02), higher education (β=.42; *P*<.001), better socioeconomic status (β=.26; *P*=.001), stronger social support (β=.17; *P*=.03), and more depressive symptoms (β=.18; *P*=.02). Among users, engagement frequency showed no significant average change over time (mean slope=–0.07, SE 0.07; *P*=.31). Higher initial engagement was associated with higher education (β=.36; *P*<.001) and social support (β=.20; *P*=.04). Better child rehabilitation status was associated with steeper declines in engagement over time (β=–.28; *P*=.03).

**Conclusions:**

Adoption and engagement showed distinct longitudinal patterns and were associated with different factors. The findings suggest that reducing digital barriers and aligning support with caregivers’ evolving needs may be relevant to facilitating platform use and sustained engagement. This study offers a theory-informed lens for describing digital support platform use among vulnerable caregiving populations.

## Introduction

### Background

Amid rapid digitalization, family caregivers of children with disabilities are increasingly seeking support online. Substantial evidence shows high caregiving stress and poor mental health outcomes in this population [1-3]. Caregivers need reliable information about rehabilitation services, special education, and policy, as well as emotional understanding and support. However, limited professional resources and disability-related stigma often constrain offline help seeking. In this context, digital support platforms (eg, peer forums, social media groups, and specialized online communities) offer scalable, low-cost channels for information exchange and emotional support, often with anonymity [4].

While digital platforms have been shown to enhance perceived social support and improve caregiver well-being [4,5], most evidence is cross-sectional or intervention-specific. Less is known about how routine platform use changes over time and whether determinants differ for adoption vs engagement. Addressing these gaps is important for theory—clarifying how vulnerable populations engage with digital health resources—and for practice—informing design and policy that foster equitable and sustained engagement.

### Use of Digital Support Platforms Among Caregivers of Children With Disabilities

Caregivers commonly use digital platforms for 2 core functions. First, information seeking: they look for practical guidance on condition management, rehabilitation options, and special education to address day-to-day challenges [6]. Second, social and emotional support: platforms connect families facing similar circumstances, facilitating experience sharing and mutual understanding that can alleviate isolation [7,8]. Qualitative evidence indicates that platforms aligning with both informational and emotional needs foster more positive engagement [9].

Evidence for benefits spans unstructured communities and structured programs. Open online communities have been associated with higher perceived social support and lower stress levels among parents of children with autism [8]. Structured interventions (eg, CaregiverTLC) can strengthen self-efficacy and coping capacity [10,11]. Reviews suggest that peer support networks may serve as a pathway to strengthening family caregivers and fostering their resilience [12]. Studies also point to potential compensatory effects, with more diverse, supportive networks linked to lower major depression risk [13].

### Factors Associated With the Use of Digital Support Platforms

Caregivers’ digital platform use can be understood through Andersen’s behavioral model of health services use (BMHSU), which categorizes determinants into 3 domains: predisposing characteristics, enabling resources, and needs [14]. Predisposing characteristics (eg, age, gender, and education) reflect general propensities to use services. Enabling resources (eg, socioeconomic conditions, social support, and access to devices or connectivity) shape the practical feasibility of use. Needs, both perceived by individuals and evaluated by professionals, are the most proximal drivers of use. The model has demonstrated utility across traditional health services and increasingly in digital health contexts [15-17].

For caregivers, 3 key factors emerge. First, predisposing characteristics matter. Higher educational attainment is generally associated with greater use for accessing and sharing health information, while older age, lower education, and limited language proficiency are linked to barriers such as lower digital literacy and reduced access [18-21]. Gender differences in engagement remain unclear, as studies often overrepresent mothers and reflect caregiving roles [4,20].

Second, enabling resources are pivotal. Socioeconomic status—income, employment, and access to devices and connectivity—conditions capacity to engage and shapes use patterns [22]. Contextual enabling conditions, including credible information and robust privacy protections, can lower perceived risk; caregivers often prefer closed groups to safeguard privacy [23,24]. Social support further enables platform use by providing informational, emotional, and practical assistance, which helps caregivers navigate digital tools and sustains engagement [25,26].

Third, needs are immediate drivers of use. Psychological distress often drives online support seeking, with higher depressive symptoms linked to greater pursuit of emotional support [27-29]. Children’s health and rehabilitation status represent key evaluated needs that sustain information seeking and support seeking over time. Even after receiving a diagnosis, many parents continue to consult multiple sources for condition details, prognosis, and management strategies, highlighting the persistent role of clinical needs in motivating digital engagement [30].

### The Present Study

Despite growing interest in caregivers’ use of digital support platforms, the literature is dominated by cross-sectional designs and short-term intervention evaluations that seldom track routine, real-world use over time. Consequently, evidence on sustained use beyond program contexts remains limited. Prior work rarely distinguishes whether caregivers use a platform (adoption) from how often they participate among users (engagement). Conflating these processes introduces zero-inflation concerns and may obscure distinct trajectories and determinants.

To address these gaps, we conducted a 3-wave, 6-month longitudinal study of 240 family caregivers of children with disabilities in China. We used a 2-part latent growth curve model to jointly estimate adoption (use vs nonuse) and engagement (frequency among users) trajectories [31,32]. This approach accommodates zero-inflated, semicontinuous data and enables direct comparison of predictors across processes.

**Figure 1.**
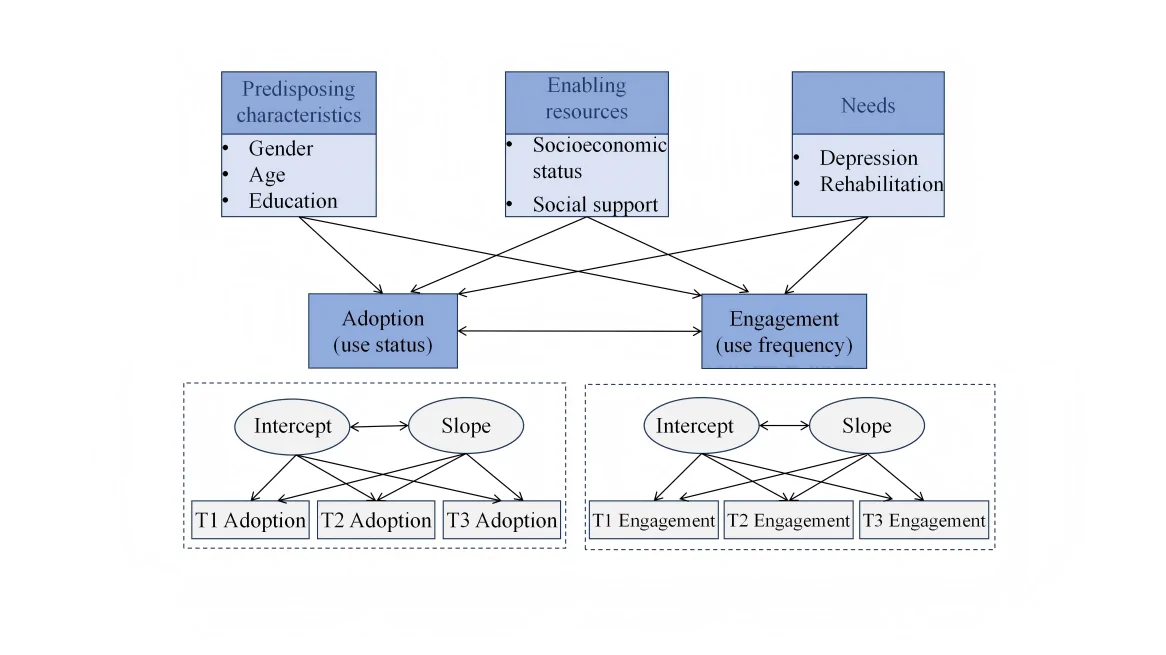
Conceptual framework of the study.

Guided by the BMHSU [14] and tailored to the caregiving context, we examined predictors in 3 domains: predisposing characteristics (caregiver gender, age, and education), enabling resources (socioeconomic status and social support), and needs (caregivers’ depressive symptoms as perceived need; children’s rehabilitation status as evaluated need). The conceptual framework is presented in Figure 1.

We hypothesized that both adoption probability and engagement would increase over time (hypothesis 1), there would be significant interindividual variability in the initial levels and growth trajectories of both adoption and engagement (hypothesis 2), and predictors would differentially relate to adoption vs engagement (hypothesis 3).

## Methods

### Study Design

This study used a prospective longitudinal design with 3 survey waves. Digital support platform use was repeatedly measured at each wave using a 3-month recall window aligned with the interwave interval. All other predictors, including demographics and psychosocial variables, were assessed at baseline and used in analyses as predictors.

### Participants

The inclusion criteria were as follows: (1) being the primary family caregiver (ie, the adult most responsible for the child’s daily care and health decisions) of a child or adolescent aged ≤18 years with a confirmed disability diagnosis, (2) ability to complete an online smartphone survey, and (3) provision of electronic informed consent. The exclusion criteria were as follows: (1) severe cognitive or communication barriers precluding survey completion and (2) duplicate entries within the same household (1 caregiver per child).

We recruited caregivers from rehabilitation centers, special education schools, and hospitals in Sichuan, Chongqing, and Shandong, China. A total of 485 valid wave 1 responses were obtained; 259 (53.4%) caregivers consented to be recontacted. Of these, 199 and 191 valid responses were collected at waves 2 and 3, respectively. The longitudinal analytic sample comprised 240 caregivers who completed at least 1 follow-up wave. Because recruitment relied on convenience sampling via advertisements and voluntary self-registration, the number of individuals who were exposed to the recruitment materials or invited to participate was not available; therefore, a formal response rate could not be calculated.

Participant characteristics were as follows: 29% (70/240) were male, with a mean age of 37.29 (SD 6.85) years. Most (215/240, 89.6%) had completed junior high school or higher, and 33% (78/240) held a bachelor’s degree or above. Subjective socioeconomic status was rated as average or below by 86.3% (207/240). The most common child disabilities were intellectual (65/240, 27%) and psychiatric (51/240, 21%). Rehabilitation status was mostly rated as fair or good (174/240, 72.7%). Additional details are presented in Table S1 in Multimedia Appendix 1.

### Measures

#### Digital Support Platform Use

A study-developed measure assessed digital support platform use, informed by prior interviews with family caregivers to ensure cultural relevance and alignment with common platform formats. The questionnaire was reviewed by 2 subject matter experts before data collection. To reduce recall bias, respondents reported their platform use over the past 3 months, with descriptions and examples provided for each platform type. Participants indicated how often they used disability-focused digital support platforms (eg, service portals, live streams, chat rooms, social media groups, and online forums) in the past 3 months. Responses were coded as 0=never, 1=rarely (eg, less than once per month), 2=occasionally (eg, 1‐3 times per month), 3=often (eg, 1‐3 times per week), and 4=almost daily (eg, 4 or more times per week). Platform adoption was coded as 0=nonuse and 1=any use (responses 1‐4), while engagement was modeled as the frequency of use among users (responses 1‐4). Nonusers were asked to report reasons for nonuse, including lack of time, unawareness, unfamiliarity, privacy concerns, or other (open ended).

#### Social Support

Perceived social support was measured at baseline using the 12-item Social Support Scale [33], which assesses family, friend, and other support. Items are rated on a 7-point scale (1=strongly disagree to 7=strongly agree). According to the scoring criteria in the original guidance, total scores of 12 to 36, 37 to 60, and 61 to 84 indicate low, moderate, and high support, respectively. In the present study, the Cronbach α for the scale was 0.92.

### Depression

Depressive symptoms were measured at baseline with the Chinese version of the 9-item Patient Health Questionnaire [34,35]. The scale consists of 9 items rated on a 4-point scale (0=not at all to 3=nearly every day). Scores of 5, 10, 15, and 20 correspond to the cutoff points for mild, moderate, moderately severe, and severe depressive symptoms, respectively. The Chinese version of this scale has been previously validated in a sample of parents of children with disabilities in China [36]. In the present study, the Cronbach α for the scale was 0.94.

### Demographic Characteristics

Demographic characteristics were collected at baseline for both the child and the caregiver. Child factors included the primary disability type (visual, hearing, physical, speech, intellectual, psychiatric, or multiple) and current rehabilitation status rated on a 5-point scale (1=extremely poor to 5=excellent). Caregiver factors included gender, age, education, and subjective socioeconomic status, obtained via a structured questionnaire.

### Ethical Considerations

This study adhered to the ethical principles of the Declaration of Helsinki and was approved by the Academic Committee of Southwest Minzu University (SMU-202401037). All participants provided electronic informed consent prior to participation.

### Procedures

The study was conducted from September 2024 to July 2025, with 3 waves of data collection at 3-month intervals. Participants were recruited through collaborating schools, hospitals, and rehabilitation centers in Sichuan Province, Chongqing Municipality, and Shandong Province. These institutions distributed posters advertising the study, and interested caregivers of children with disabilities voluntarily registered by scanning a QR code. Eligible participants who provided informed consent were invited to complete a 10-minute online survey for each wave. To prevent duplicate submissions, IP-based restrictions were implemented, and surveys completed in unrealistically short times were excluded as invalid. Follow-up surveys were distributed 3 months after the participants’ initial survey date. As an incentive, participants received CNY 10 (CNY 1=US $0.15 as of September 5, 2026) for each completed wave.

### Data Analysis

Analyses included participants who completed the baseline and at least 1 follow-up assessment (n=240). To examine selective attrition, baseline characteristics were compared between participants retained for longitudinal analyses and those lost to follow-up. No significant differences were found in platform use (*χ*²_4_=7.5; *P*=.11), age (*t*_483_=0.57; *P*=.57), education (*χ*²_5_=5.6; *P*=.35), subjective socioeconomic status (*χ*²_4_=5.3; *P*=.26), disability type (*χ*²_6_=9.9; *P*=.13), child rehabilitation status (*χ*²_4_=8.2; *P*=.08), or depression (*t*_483_=–1.20; *P*=.23). However, the attrition group had a higher proportion of men (*χ*²_1_=7.2; *P*<.01) and higher social support (*t*_483_=3.97; *P*<.001). Overall, selective attrition appeared modest: most baseline characteristics did not differ, although men and those with higher social support were more likely to drop out. Missing data were handled using full information maximum likelihood (FIML).

Descriptive statistics and bivariate correlations were computed for key variables. Core analyses used a 3-wave, 2-part latent growth curve model (time coded as 0, 1, and 2) to decompose platform behavior into two components: (1) adoption (any use vs nonuse; categorical probit specification; latent intercept *I_B_* and slope *S_B_*) and (2) engagement (use frequency among users; measured on a 4-level ordinal scale treated as continuous in the primary model; latent intercept *I_C_* and slope *S_C_*). Random intercepts and slopes were allowed to covary within and across components to account for shared determinants and correlated dynamics. Engagement was reestimated as ordinal for robustness, yielding consistent results.

Unconditional models were first estimated to assess mean trajectories and variance components. Covariates (gender, age, education, socioeconomic status, social support, depressive symptoms, and child rehabilitation status) were then added to predict the 4 growth factors. All models used maximum likelihood estimation with robust standard errors in Mplus (version 7.4; Muthén & Muthén). Model fit was evaluated using chi-square statistics, Akaike information criterion (AIC), Bayesian information criterion (BIC), and sample size–adjusted BIC. Robustness checks were performed by reestimating the model with the full baseline sample (n=485) using FIML to handle longitudinal missingness across waves.

## Results

### Digital Platform Use Among Caregivers Across 3 Waves

Across 3 waves, caregivers reported how often they had used any disability-related digital platform in the past 3 months; nonusers additionally selected one or more reasons for nonuse. Table 1 presents the distributions of use frequency and reasons for nonuse at each wave.

**Table 1.** The use of disability-related digital platforms among caregivers.^a^

	T1^b^, n (%)	T2^b^, n (%)	T3^b^, n (%)
Use frequency			
Never	65 (27)	47 (24)	27 (14)
Rarely	23 (10)	16 (8)	19 (10)
Occasionally	56 (23)	44 (22)	54 (28)
Often	58 (24)	66 (33)	65 (35)
Almost daily	38 (16)	26 (13)	26 (13)
Reasons for nonuse			
Limited time or energy	22 (34)	13 (28)	6 (24)
Unaware of platform availability	40 (62)	20 (44)	7 (28)
Unfamiliar with how to use platforms	4 (6)	15 (33)	10 (40)
Concerns about cybersecurity or privacy	6 (9)	10 (22)	1 (4)

a Percentages are based on respondents with nonmissing data at each wave. Nonuse reasons were multiple response items.

b T1 represents the first measurement, T2 represents the second measurement, and T3 represents the third measurement.

Descriptively, the share of caregivers reporting any use increased over time, reflected in a decline in “Never” from 27% (65/240) to 14% (27/191). The distribution also shifted toward midrange categories (“Occasionally” and “Often”), whereas the proportion reporting “Almost daily” was relatively stable, with a slight decrease from T1 to T3.

Regarding nonuse, lack of awareness of platform availability and limited time or energy were the most frequently cited barriers at T1. Over time, reports of awareness and time or energy barriers declined, whereas unfamiliarity with how to use platforms increased, suggesting a shift from access or awareness constraints toward competence-related barriers. Cybersecurity or privacy concerns peaked at T2 and declined at T3.

### Descriptive Statistics and Correlations

Descriptive statistics and bivariate associations among study variables across the 3 waves are summarized in Table 2. Spearman rank correlations were used to accommodate ordinal scaling and potential nonnormality. Platform-use categories exhibited moderate temporal stability, and associations with covariates varied in magnitude and direction.

**Table 2.** Descriptive statistics and correlations of study variables.

Variables	FRE1^a^^,^^b^	FRE2^c^	FRE3^d^	GEN^e^^,^^f^	AGE^g^	EDU^h^^,^^i^	SES^j^^,^^k^	REC^l^^,^^m^	SS^n^^,^^o^	DEP^p^^,^^q^
FRE 1										
*r* value	1									
*P* value	—^r^									
FRE 2										
*r* value	0.60	1								
*P* value	<.001	—								
FRE 3										
*r* value	0.51	0.59	1							
*P* value	<.001	<.001	—							
GEN										
*r* value	0.19	0.22	0.21	1						
*P* value	.003	.001	.004	—						
AGE										
*r* value	−0.17	−0.22	−0.15	−0.16	1					
*P* value	.01	.002	.04	.02	—					
EDU										
*r* value	0.48	0.39	0.39	0.19	−0.29	1				
*P* value	<.001	<.001	<.001	.003	<.001	—				
SES										
*r* value	0.30	0.32	0.22	0.18	−0.19	0.34	1			
*P* value	<.001	<.001	.002	.005	.003	<.001	—			
REC										
*r* value	0.12	0.01	0.01	0.04	0.02	0.12	0.14	1		
*P* value	.06	.84	.87	.58	.71	.08	.03	—		
SS										
*r* value	0.08	0.28	0.14	0.20	−0.06	−0.03	0.16	0.07	1	
*P* value	.21	<.001	.05	.002	.39	.68	.01	.26	—	
DEP										
*r* value	0.01	0.04	−0.04	−0.02	0.06	−0.07	−0.19	0.01	−0.22	1
*P* value	.82	.59	.56	.78	.36	.32	.004	.93	.001	—

a FRE: frequency use (T1-T3).

b Mean 1.92 (SD 1.43).

c Mean 2.04 (SD 1.37).

d Mean 2.23 (SD 1.21).

e GEN: gender (0=female, 1=male).

f Mean 0.29 (SD 0.46).

g Mean 37.29 (SD 6.85).

h EDU: education level.

i Mean 3.48 (SD 1.45).

j SES: socioeconomic status.

k Mean 2.55 (SD 0.95).

l REC: child rehabilitation status.

m Mean 2.97 (SD 0.80).

n SS: social support.

o Mean 59.65 (SD 13.60).

p DEP: depression.

q Mean 10.56 (SD 6.71).

r Not applicable.

### Two-Part Latent Growth Analysis of Digital Platform Use and Predictors

To distinguish between adoption and engagement intensity, platform use was modeled using a 2-part latent growth curve model. The binary component represented use status at each wave (b1-b3), while the continuous component captured use frequency among users (c1-c3). Unconditional growth models were first estimated separately for each part and then combined in a parallel 2-part model. In preliminary analyses, allowing the adoption slope variance (*S_B_*) to be freely estimated resulted in numerical instability and nonconvergence, indicating insufficient information to reliably estimate between-person variability in change in the 3-wave binary adoption process. Therefore, in the primary models, we fixed Var(*S_B_*)=0, retained a random intercept for adoption, and estimated the mean adoption slope to characterize the average change in adoption over time. The unconditional 2-part model showed acceptable fit (Pearson *χ*²_4_=2.2, *P*=.69; likelihood ratio *χ*²_4_=2.4, *P*=.67; AIC=1805.95; BIC=1851.19; adjusted BIC=1809.99).

We then estimated a conditional 2-part model incorporating gender, age, education, socioeconomic status, child rehabilitation status, social support, and caregiver depressive symptoms. Compared with the unconditional model, the conditional model showed lower AIC (1736.54 vs 1805.95) and sample size–adjusted BIC (1747.11 vs 1809.99), although BIC increased slightly (1854.88 vs 1851.19). Given the a priori inclusion of covariates and overall model performance, we retained the conditional model for inference.

In the binary part, the mean slope was positive (*S_B_*=0.54, SE 0.16; *P*=.001), indicating increasing probability of adoption over time; the intercept variance was significant (Var(*I_B_*)=2.76, SE 0.82; *P*=.001), evidencing substantial heterogeneity at baseline. In the continuous part, the mean intercept indicated relatively high initial frequency among users with significant between-person variability (*I_C_*=2.52, SE 0.10; *P*<.001; and Var(*I_C_*)=0.58, SE 0.14; *P*<.001). The mean slope was not significant (*S_C_*=−0.07, SE 0.07; *P*=.31), but its variance approached significance (Var(*S_C_*)=0.11, SE 0.06; *P*=.07), indicating variability in individual change rates. A strong negative correlation between the intercept and slope (*r*=−0.72; *P*<.001) suggested that users with higher initial use frequency tended to show flatter or declining trajectories over time.

Covariate effects indicated that education was a robust positive correlate of baseline adoption, with additional positive associations for socioeconomic status, social support, depressive symptoms, and male gender. For initial use frequency among users, education and social support were positive predictors, whereas other effects were small and nonsignificant. Regarding change in frequency, better child rehabilitation status predicted steeper declines over time. Parameter estimates are presented in Tables 3 and 4.

**Table 3.** Estimated means and variances of growth factors in the 2-part latent growth curve model.^a^

Latent factor	Growth factors, mean (SE)	*P* value	Growth factors, variance (SE)	*P* value
Use status intercept (*I_B_*)	N/A^b^	N/A	2.76 (0.82)	.001
Use status slope (*S_B_*)	0.54 (0.16)	.001	0 (fixed)	N/A
Use frequency intercept (*I_C_*)	2.52 (0.10)	<.001	0.58 (0.14)	<.001
Use frequency slope (*S_C_*)	−0.07 (0.07)	.31	0.11 (0.06)	.07

a The variance of *S*_*B*_ was fixed to zero due to numerical instability and nonconvergence when freely estimated. The mean of *I*_*B*_ is not separately estimated under the categorical (threshold or latent response, probit) parameterization.

b N/A: not applicable.

**Table 4.** Predictors of adoption intercept and engagement growth factors.^a^

Predictor	Adoption intercept *I_B_*, β (SE)	*P* value	Engagement intercept *I_C_*, β (SE)	*P* value	Engagement slope *S_C_*, β (SE)	*P* value
Gender	0.19 (0.08)	.02	−0.04 (0.08)	.68	0.12 (0.12)	.34
Age (z-score)	−0.11 (0.07)	.11	0.00 (0.08)	.96	−0.12 (0.14)	.37
Education (z)	0.42 (0.08)	<.001	0.36 (0.10)	<.001	0.04 (0.15)	.78
Socioeconomic status (z)	0.26 (0.08)	.001	0.11 (0.09)	.21	−0.05 (0.14)	.69
Rehabilitation (z)	−0.08 (0.09)	.37	0.13 (0.08)	.10	−0.28 (0.13)	.03
Social support (z)	0.17 (0.08)	.03	0.20 (0.10)	.04	−0.09 (0.15)	.55
Depression (z)	0.18 (0.08)	.02	0.08 (0.08)	.31	−0.02 (0.12)	.89

a Standardized coefficients (β) with SEs and *P* values. IB uses a categorical probit specification. SB was not regressed on covariates because Var(*S*_*B*_) was fixed to 0.

As a sensitivity analysis, we reestimated the 2-part model with the full baseline sample (n=485) using FIML to handle longitudinal missingness across waves. Results were largely consistent with the primary analysis, supporting the robustness of the main conclusions. Caregiver age became a significant predictor of baseline adoption in the full baseline sample, whereas the rehabilitation–engagement growth association was attenuated to a marginal level (directionally consistent). Detailed estimates are provided in Tables S2 and S3 in Multimedia Appendices 2 and 3, respectively.

## Discussion

This study used a 2-part latent growth curve model to explore the dynamics and potential predictors of digital support platform use among family caregivers of children with disabilities. Findings indicated that platform adoption increased over time, while average engagement remained stable. Distinct predictors for adoption and engagement may reflect differing processes underlying initial uptake vs sustained participation.

### Status and Trajectory of Digital Platform Use

At the adoption level, the positive mean slope in the binary part suggests an increasing probability of platform use over time, with adoption becoming more common. This increase may reflect the growing availability of online resources and broader shifts toward digital help seeking [37]. Notably, the slope variance (*S_B_*) was not stably estimable and was therefore fixed to zero. Consequently, between-person variability in adoption change over time was not modeled, which may underestimate heterogeneity in adoption trajectories.

A different pattern emerged for engagement frequency. Baseline engagement frequency (*I_C_*) showed significant between-person variability, while the mean slope (*S_C_*) was near zero, indicating stable average engagement at the group level. Evidence for variability in *S_C_* was marginal, suggesting some heterogeneity in individual trajectories. The negative correlation between *I_C_* and *S_C_* suggests differing trends by initial engagement level, possibly reflecting needs-based adjustment, regression to the mean, or ceiling effects.

### Factors Associated With Digital Support Platform Use Among Caregivers

Guided by the Andersen BMHSU [14], we examined predisposing (gender, age, and education), enabling (subjective socioeconomic status and social support), and need-related (child rehabilitation status and depressive symptoms) factors.

For adoption, education emerged as a robust positive correlate, consistent with evidence linking higher educational attainment to digital literacy and earlier uptake [20]. Subjective socioeconomic status was also positively associated with adoption, aligning with research highlighting the role of digital capital and reduced barriers among higher-status groups [26,38]. Social support was another significant predictor, indicating that richer offline networks may facilitate online navigation [25]. Depressive symptoms were positively associated with adoption, supporting the notion of need-driven help-seeking behavior [29].

Interestingly, in this sample, male caregivers had a higher baseline probability of adoption, contrasting with prior studies that often report greater digital use among females [39]. However, this finding may reflect study-specific factors such as caregiver demographics, selection bias, or attrition and should not be overinterpreted as evidence of a stable gender difference. Instead, it highlights the context-dependent nature of gender effects on platform use, which may vary across settings.

For engagement frequency, only education and social support predicted baseline levels, suggesting that engagement is more closely tied to competencies and social resources than to other sociodemographic characteristics. Changes in engagement frequency were most consistently related to child rehabilitation status: better rehabilitation outcomes tended to coincide with steeper declines in use, consistent with Andersen’s premise that lower perceived need is linked to reduced use [14,16]. However, this association was attenuated to a marginal level in the full baseline sample, and alternative explanations (eg, unmet evolving needs among families with improving outcomes) should be considered.

Overall, the findings partially support Andersen’s model for understanding caregivers’ digital platform use. At the same time, digital health use often unfolds in 2 stages—adoption and engagement—and may also depend on technology-specific mechanisms. The technology acceptance model (TAM) and unified theory of acceptance and use of technology (UTAUT) emphasize perceived usefulness, ease of use, and facilitating conditions (eg, skills, support, and resources) [40,41], while privacy-trust research highlights privacy risk appraisal and trust [42]. Although we did not directly measure these constructs, descriptively reported barriers changed over time: “lack of awareness” declined from T1 to T3, “not knowing how to use” increased, and privacy concerns peaked at T2 but declined by T3. These patterns suggest that integrating TAM or UTAUT and privacy-trust constructs with Andersen’s framework may better capture the dynamics of adoption and sustained engagement in digital health contexts.

### Practical Implications

On the basis of these findings, several practical implications emerge. To support adoption and reduce inequities, interventions may prioritize caregivers with lower educational attainment, socioeconomic status, and social support. Simplified onboarding delivered through community networks, schools, and rehabilitation centers may lower entry barriers, and brief digital literacy training could address capability gaps. Targeted awareness campaigns may further promote adoption. Because adoption was higher among caregivers reporting more depressive symptoms, onboarding should also include clear pathways to emotional and psychological support, including referral options.

To support sustained engagement, digital health platforms should be adaptive, user-friendly, and trustworthy. Given stable average engagement but heterogeneous individual trajectories, personalization can adjust content, peer matching, and support intensity as caregiver needs change [43,44]. AI-based guidance may help reduce information overload and improve perceived usefulness, but implementation requires appropriate governance and safeguards [45,46]. Usability can be strengthened through caregiver-centered interface design [47], and privacy protections with transparent communication are important for maintaining trust [48].

Finally, integrating digital platforms into existing support systems may enhance reach and sustainability. Coordinated efforts across government agencies, social welfare organizations, rehabilitation centers, and special education schools can support promotion, onboarding, linkage to services, and ongoing feedback so that platforms evolve with caregivers’ needs.

### Limitations

This study has several limitations. First, platform use was measured with a study-specific self-report instrument informed by caregiver interviews and expert review; however, the instrument has not undergone formal psychometric validation, including evaluation of construct validity, criterion validity, and reliability. Engagement was also operationalized only as use frequency, without capturing other dimensions such as session intensity, interaction quality, or emotional engagement. Consequently, qualitatively different use patterns (eg, frequent passive browsing vs infrequent but high-quality interaction) may have been conflated, limiting interpretation of engagement trajectories.

Second, although multicenter recruitment may reduce regional bias, convenience sampling may have favored caregivers with higher needs, limiting representativeness. Differential attrition (with higher dropout among male participants and those with greater baseline social support) may further bias estimates. Attrition also reduced sample size and power for detecting longitudinal effects, so null findings should be interpreted cautiously.

Third, because participants were recruited primarily in China, generalizability to other contexts is uncertain. Cross-country differences in caregiving systems, digital literacy, internet access, and health care infrastructure may shape adoption and engagement.

Finally, although the longitudinal design supports temporal ordering, the study remains observational and measurement reactivity cannot be ruled out; completing baseline assessments may have influenced subsequent platform use. Future studies could lessen potential priming from surveys by shortening assessments and, where possible, supplementing self-report with platform use logs.

### Conclusions

This study used a 2-part latent growth curve model to examine digital platform use among caregivers of children with disabilities in China. Adoption increased over time and was positively associated with education, gender, subjective socioeconomic status, social support, and depressive symptoms. Average engagement remained stable, with baseline engagement related to education and social support. Changes in engagement were most consistently associated with child rehabilitation status. Overall, these findings underscore the value of distinguishing adoption from engagement and suggest that caregiver resources and needs are related to patterns of digital platform use.

## Supplementary material

10.2196/92481 Multimedia Appendix 1 Demographic characteristics of participants.

10.2196/92481 Multimedia Appendix 2 Estimated means and variances of growth factors in the 2-part latent growth curve model in the full baseline sample.

10.2196/92481 Multimedia Appendix 3 Predictors of adoption intercept and engagement growth factors in the full baseline sample.

## Abbreviations

AIC: Akaike information criterion
BIC: Bayesian information criterion
BMHSU: behavioral model of health services use
FIML: full information maximum likelihood
TAM: technology acceptance model
UTAUT: unified theory of acceptance and use of technology
