# Supplementary material for: Dynamics of Digital Support Platform Use Among Family Caregivers of Children With Disabilities: Longitudinal Study
Source: JMIR Mhealth Uhealth. 2026 Sep 23;14:e92481. doi: 10.2196/92481 (PMC13600590; doi:10.2196/92481)
Supplement: Supplementary file 1 [file mhealth-v14-e92481-s001.docx]

**Multimedia Appendix 1.**

Table S1. Demographic Characteristics of Participants

|  | Categories | *n* (%) |
| --- | --- | --- |
|  |  |  |
| Gender | Male | 70 (29) |
|  | Female | 170 (70.8) |
| Education level | 1 Primary school or below | 25 (10) |
|  | 2 Junior high school | 48 (20) |
|  | 3 Senior high school / Technical secondary school / Vocational school | 43 (18) |
|  | 4 Associate degree | 46 (19) |
|  | 5 Bachelor’s degree | 68 (28) |
|  | 6 Master’s degree or above | 10 (4) |
| Subjective family socioeconomic status | 1 Extremely poor | 30 (13) |
|  | 2 Poor | 89 (37) |
|  | 3 Average | 88 (37) |
|  | 4 Good | 26 (11) |
|  | 5 Excellent | 7 (3) |
| Types of disability | 1 Visual disability | 16 (7) |
|  | 2 Hearing disability | 31 (13) |
|  | 3 Physical disability | 25 (10) |
|  | 4 Speech disability | 26 (11) |
|  | 5 Intellectual disability | 65 (27) |
|  | 6 Psychiatric disability | 51 (21) |
|  | 7 Multiple disabilities | 26 (11) |
| Rehabilitation status | 1 Extremely poor | 7 (3) |
|  | 2 Poor | 58 (24) |
|  | 3 Fair | 112 (46.7) |
|  | 4 Good | 62 (26) |
|  | 5 Excellent | 1 (0.4) |
