# Supplementary material for: Dynamics of Digital Support Platform Use Among Family Caregivers of Children With Disabilities: Longitudinal Study
Source: JMIR Mhealth Uhealth. 2026 Sep 23;14:e92481. doi: 10.2196/92481 (PMC13600590; doi:10.2196/92481)
Supplement: Supplementary file 2 [file mhealth-v14-e92481-s002.docx]

**Multimedia Appendix 2.**

Table S2. Estimated Means and Variances of Growth Factors in the Two-Part Latent Growth Curve Model in the Full Baseline Sample

| Latent factor | Mean | *SE* | *P* | Variance | *SE* | *P* |
| --- | --- | --- | --- | --- | --- | --- |
| Usage Status Intercept (*I_B_*) | NA | NA | NA | 2.88 | 0.85 | .001 |
| Usage Status Slope (*S_B_*) | 0.56 | 0.16 | .001 | 0 (fixed) | NA | NA |
| Usage Frequency Intercept (*I_C_*) | 2.57 | 0.09 | <.001 | 0.53 | 0.12 | <.001 |
| Usage Frequency Slope (*S_C_*) | -0.08 | 0.06 | .18 | 0.11 | 0.06 | .05 |

Note. NA = not applicable/not estimated. The variance of *S_B_* was fixed to zero due to numerical instability and non-convergence when freely estimated. The mean of *I_B_* is not separately estimated under the categorical (threshold/latent-response, probit) parameterization.
