# Supplementary material for: Dynamics of Digital Support Platform Use Among Family Caregivers of Children With Disabilities: Longitudinal Study
Source: JMIR Mhealth Uhealth. 2026 Sep 23;14:e92481. doi: 10.2196/92481 (PMC13600590; doi:10.2196/92481)
Supplement: Supplementary file 3 [file mhealth-v14-e92481-s003.docx]

**Multimedia Appendix 3.**

Table S3. Predictors of Adoption Intercept and Engagement Growth Factors in the Full Baseline Sample

| Predictor | Adoption intercept *I_B:_*  *β* (SE) | *P* | Engagement intercept *I_C_*:  *β* (SE) | *P* | Engagement slope *S_C_* :  *β*(SE) | *P* |
| --- | --- | --- | --- | --- | --- | --- |
| Gender | 0.14 (0.07) | .04 | −0.01 (0.06) | .88 | 0.11 (0.16) | .47 |
| Age (z) | −0.16 (0.06) | .009 | −0.01 (0.07) | .94 | −0.14 (0.13) | .28 |
| Education (z) | 0.44 (0.06) | <.001 | 0.29 (0.07) | <.001 | 0.15 (0.13) | .24 |
| SES (z) | 0.26 (0.07) | <.001 | 0.10 (0.07) | .15 | −0.03 (0.12) | .79 |
| Rehabilitation (z) | −0.02 (0.07) | .76 | 0.07 (0.06) | .24 | −0.20 (0.11) | .07 |
| Social support (z) | 0.22 (0.06) | .001 | 0.24 (0.07) | <.001 | −0.10 (0.12) | .40 |
| Depression (z) | 0.18 (0.07) | .007 | 0.01 (0.06) | .94 | 0.07 (0.11) | .55 |

Note. STDYX standardized coefficients (*β*) with SEs and *P* values. IB uses a categorical probit specification. SB was not regressed on covariates because Var(*S_B_*) was fixed to 0.
